# Acupuncture for patients recovering from lacunar infarction: A protocol for systematic review and meta-analysis

**DOI:** 10.1097/MD.0000000000026413

**Published:** 2021-06-25

**Authors:** Haoran Wang, Xiaoyan Fu, Jing Ju, Dan Meng, Shengming Sun, Chenchen Guo, Hongling Jia, Qiangsan Sun

**Affiliations:** a Department of Rehabilitation Medicine, The Second Hospital of Shandong University; b Department of Endocrinology, Jinan Huaiyin People's Hospital; c Shandong University of Traditional Chinese Medicine; d Department of Traditional Chinese Medicine, Shandong Provincial Public Health Clinical Center; e Neck-Shoulder and Lumbocrural Pain Hospital Affiliated to Shandong First Medical University; f The Second Affiliated Hospital of Shandong University of Traditional Chinese Medicine, Jinan, Shandong, China.

**Keywords:** acupuncture, lacunar infarction, meta-analysis, protocol, recovery period, systematic review

## Abstract

**Background:**

Lacunar infarction (LI) is the mild type in the classification of ischemic stroke, mostly occurs in the middle-aged and elderly, with mild hemiplegia and partial sensory disorder as the main manifestations. In the treatment of LI, acupuncture is often regarded as dominant therapy in the convalescence period. However, acupuncture for treatment of LI in the recovery period lacks high-quality reports and evidence-based medical evidence. Thus, we aim to evaluate the curative effect and safety of acupuncture for LI objectively.

**Methods:**

Pubmed, Cochrane Library, Web of Science, EBSCO, Springer, China National Knowledge Infrastructure, Chinese Scientific and Technical Journals Database (VIP), Wan-fang Database, Chinese Biomedical Literature Database, Chinese Science Citation Database, and other electronic databases will be retrieved from the inception to May, 2021. Randomized controlled trials related to this subject will be searched. The inclusion criteria are established and a detailed literature search strategy is designed through discussion. Article retrieval, screening, excluding repetitive studies, assessment of quality, and data processing will be conducted by 2 reviewers independently using EndNote (X9) and Review Manager (5.3.5). The outcome measures include primary outcome measures (total effective rate, National Institute of Health Stroke Scale score, and Fugl-Meyer Assessment score), secondary outcome measures (blood pressure, plasma glucose, and blood lipid), and safety outcome measures. We will perform a meta-analysis, descriptive analysis, and subgroup analysis based on data conditions.

**Results:**

The study of total effective rate, National Institute of Health Stroke Scale score, Fugl-Meyer Assessment score, blood pressure, plasma glucose, blood lipid, and adverse effects will provide evidenced outcome for high-quality synthesis and descriptive analysis.

**Conclusion:**

This systematic review will kindly provide evidence of whether acupuncture is an effective and safe intervention for LI in the recovery period.

**INPLASY registration number:**

INPLASY202150060 (DOI:10.37766/inplasy2021.5.0060).

## 1 Introduction

### 1.1 Description of the condition

As is known to all, stroke is the second leading cause of death in the world. Lacunar infarcts are small infarcts (2–20 mm in diameter) in the deep cerebral white matter, basal ganglia, or pons, account for about 25% of ischemic strokes, and they are named for their ability to form and cavitate ponds and “little lakes.”^[1–4]^ Due to the involvement of small penetrating cerebral arteries, lacunes usually result from cerebral microatheromatosis and lipohyalinosis.^[5]^ While smaller in size and the prognosis of the disease is good, if the risk factors of cerebrovascular disease are not controlled in time, it will cause repeated attacks of the disease, causing a series of chain reactions such as disabled, demented, pseudobulbar paralysis, and cognitive dysfunction.^[6]^ Traditionally, lacunar infarction (LI) has been considered a small-artery disease.^[7,8]^ Hypertension is the single most prevalent and powerful high-risk factor for stroke, particularly for stroke closely associated with cerebral small vessel disease.^[9]^ In addition, hyperglycemia and hyperlipidemia are also related to this disease. It is important to emphasize that reducing blood pressure is the key to the treatment of this disease for patients with lacunar infarcts. Therefore, to reduce the recurrence of LI and improve the symptoms of patients, appropriate prevention and treatment interventions are needed in the treatment.

### 1.2 Description and function of intervention

Acupuncture is a characteristic therapy of traditional Chinese medicine, which mainly studies meridian acupoints, acupuncture methods, and treatment rules. As acupuncture continues to develop and spread, it is becoming more and more recognized and popular around the world. Due to the advantages of wide indications, obvious efficacy, economic safety, acupuncture has been widely applied to the clinical treatment of LI. A clinical study found that acupuncture combined with Buyang Huanwu decoction could improve the clinical outcomes and reduce the recurrence rates in patients with ischemic stroke.^[10]^ In Another study, scalp acupuncture could treat ischemic stroke by improving cerebral blood circulation to promote regional energy metabolism and upregulating expression of glial cell-line-derived neurotrophic factor to promote proliferation and differentiation of neural stem cells in the focal cerebral cortex and hippocampus, etc.^[11]^ An experiment conducted in cerebral ischemia models showed that electroacupuncture regulated the activation of microglia and microglia-mediated inflammation after cerebral ischemia, confirming the relevant theories related to the effect of acupuncture treatment on cerebral ischemia.^[12]^

### 1.3 Why the systematic review is necessary

As a subtype of ischemic stroke, LI is pathogenetically most often associated with cerebral microangiopathy and makes the development of cognitive impairment up to the development of vascular dementia.^[13]^ In the clinical treatment of lacunar cerebral infarction, intravenous thrombolytic therapy, interventions for risk factors (lowering blood pressure, lowering blood glucose, lowering blood lipid) are often recommended.^[14,15]^ Although the proportion of the application of acupuncture for LI is also on the rise, there is no systematic review of acupuncture on this disease. Therefore, we will systematically evaluate the clinical efficacy and safety of acupuncture in the treatment of LI to lay a solid foundation for clinical practice.

## 2 Methods

The systematic review has been registered on the INPLASY website, and the registration number is INPLASY202150060 (DOI:10.37766/inplasy2021.5.0060). This research will adhere to the guidelines of Preferred Reporting Items for Systematic Reviews and Meta-Analysis Protocols statement.^[16]^ Each step will refer to the Cochrane Handbook (5.2.0). The purpose of this systematic review is to summarize randomized controlled trials (RCTs) of acupuncture for the treatment of LI. Fully understanding the current level of evidence from this study is the key to determining whether acupuncture is an effective intervention for patients with LI.

### 2.1 Inclusion/exclusion criteria

#### 2.1.1 Types of literature

We will only include the RCTs that are scientific and practicable with language limited to English and Chinese. In the RCTs, the treatment group will be treated with acupuncture or a combination of acupuncture and routine pharmacotherapy, and the control group will be treated with non-acupuncture treatment. Non-RCTs, reviews, duplicate publications, meta-analysis, theoretical discussion, case reports, unqualified interventions, and animal trials will be excluded from this research.

#### 2.1.2 Types of patients

We will include patients with a diagnosis of LI based on brain magnetic resonance imaging who are in the recovery period, without any limitation about age, gender, race, region, and other factors.

#### 2.1.3 Types of interventions and comparisons

The treatment group involving needle acupuncture alone or combined with therapies emerging from the control group in the treatment of LI will be included, non-needle acupuncture will be excluded. In particular, it is important to note that there are no restrictions on acupoint selection and technique. The control group in which the interventions include no treatment, placebo acupuncture, routine pharmacotherapy alone, or other active intervention. The routine pharmacotherapy contains drugs suggested by the international or domestic recognized medical guidelines. Comparisons consist of acupuncture and its relation will be excluded.

### 2.2 Types of outcomes

Primary outcomes include total effective rate, National Institute of Health Stroke Scale score, and Fugl-Meyer Assessment score. Secondary outcomes include blood pressure, plasma glucose, and blood lipid. The safety outcomes will be illustrated by the rate and seriousness of adverse events.

### 2.3 Data sources

#### 2.3.1 Electronic search strategy

PubMed, Cochrane Library, Web of Science, EBSCO, Springer, China National Knowledge Infrastructure, Chinese Scientific and Technical Journals Database (VIP), Wan-fang Database, Chinese Biomedical Literature Database, Chinese Science Citation Database , and other electronic databases will be retrieved from the inception to May 2021. Table 1 lists exemplary search strategies of PubMed with search terms matching medical subject headings. Depending on different search patterns, keywords can be combined with free words and appropriate retrieval pattern will be conducted.

**Table 1:** Search strategy for PubMed database.

Number	Strategy
#1	“Acupuncture” OR “Electroacupuncture” OR “Body acupuncture” OR “Scalp acupuncture”
#2	“lacunar infarction” OR “lacunar infarct” OR “lacunar stroke”
#3	“Randomized controlled trial” OR “Randomized” OR “Randomly” OR “Clinical trial” OR “Controlled clinical trial” OR “RCT”
#4	#1AND #2 AND #3

#### 2.3.2 Searching other resources

Baidu Scholar, Google Scholar, the US National Institutes of Health Register, WHO International Clinical Trial Registry Platform, ClinicalTrials.gov, and Chinese Clinical Trial Registry will be searched.

### 2.4 Data collection analysis

#### 2.4.1 Selection of literature

**Figure 1:**
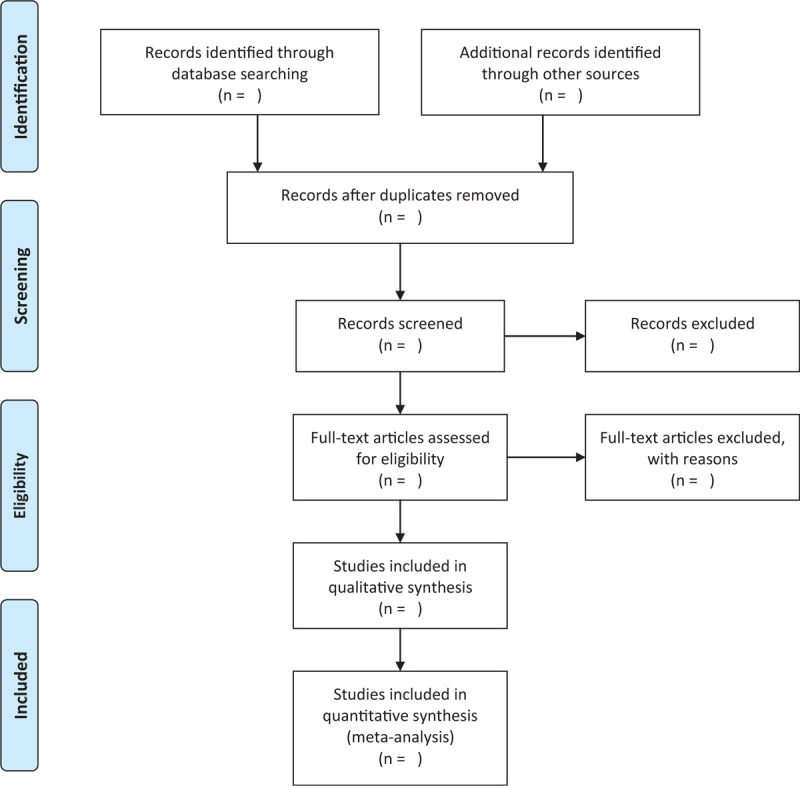
Flow chart of literature selection.

Two authors (HW and XF) will choose clinical articles based on inclusion criteria independently. After screening titles and abstracts, literature that is irrelevant, repetitive, and does not meet the criteria will be excluded by EndNote (X9). They will read the full text if necessary. The screening operation is shown in Figure 1. When the complete literature or necessary information is not available, we will try to contact the corresponding author. If there is a choice disagreement, we will consult a third-party expert (QS) to resolve it.

#### 2.4.2 Quality assessment of included literature

Based on Cochrane Handbook (5.2.0), 2 independent authors (JJ and DM) will assess the quality of included articles and the risk of bias with the Review Manager (5.3.5). Randomized methods, blinding methods, allocation concealment, completeness of outcome data, and selective reporting are all included in the quality assessment of studies. When the assessment is controversial, the opinion of a third-party expert (QS) will be sought.

#### 2.4.3 Data extraction and management

Two independent authors (HW and SS) will extract information from all eligible literature. Basic information will be extracted and compiled to Excel table: serial number, title, first author of the article, journal source, publication year, participants (number, gender, age, disease course, and pathogenic factors), interventions-related information in treatment group and control group (type, cycle, and time), outcome (total effective rate, National Institute of Health Stroke Scale score, Fugl-Meyer Assessment score, blood pressure, plasma glucose, blood lipid, and adverse events). All of the above data will be managed using Microsoft Excel 2019.

#### 2.4.4 Measures of treatment effect

Two independent authors (HW and XF) will adopt Review Manager (5.3.5) to analyze and cross-check the treatment effect. Continuous data will be expressed as mean difference or standardized mean difference with a 95% confidence interval. Risk ratio with a 95% confidence interval will be used to analyze dichotomous variables. Binary data will be converted to risk ratio format for analysis when they exist.

#### 2.4.5 Dealing with missing data

As the necessary data may be missing from the paper, the author (XF) will contact the lead author by phone or email. If no response is received, the corresponding article will be discarded because of data unavailable.

#### 2.4.6 Assessment of heterogeneity

The *Q*-test and *I*^*2*^ statistic of Review Manager (5.3.5) were used to assess the heterogeneity of data. The heterogeneity will be regarded as high (*I*^*2*^ > 75%), moderate (*I*^*2*^ between 50% and 75%), low (*I*^*2*^ <50%). It is generally believed that *I*^*2*^ ≥50% indicates substantial heterogeneity.

#### 2.4.7 Assessment of reporting bias

Funnel plots will be created to assess publication and reporting biases. A symmetric funnel plot suggests a low risk of bias, while an asymmetric funnel plot suggests a high risk of bias. When more than 10 RCTs are included in the meta-analysis, funnel plots will be created to assess reporting bias.

#### 2.4.8 Data synthesis

A meta-analysis or descriptive analysis will be performed according to the age of participants, disease course, pathogenic factors, intervention and measurement methods, and heterogeneity levels, and this like. A random-effect model will be used for the merger analysis when the heterogeneity is moderate, and a fixed-effect model will be used for the merger analysis when the heterogeneity is low. When the heterogeneity is significantly high, we will perform subgroup analysis or descriptive analysis.

#### 2.4.9 Subgroup analysis

When the results based on data synthesis indicate that this subgroup is required, we will perform the subgroup analysis. Subgroup analysis will be performed on these particular features of included literature (e.g., gender, age, disease course, pathogenic factors, interventions-related information, and measurement methods) which will cause the heterogeneity.

#### 2.4.10 Sensitive analysis

We will conduct sensitivity analysis to assess the reliability of the results. We will remove low-quality studies on an item-by-item basis and then compile the data to evaluate study quality, the impact of sample size, missing data on the results of this work, and statistical methods.

#### 2.4.11 Grading the quality of evidence

The Grading of Recommendations Assessment approach will be used to evaluate the strength of evidence for each outcome, including high, moderate, low, or very low.

#### 2.4.12 Ethics and dissemination

This protocol does not evaluate patients’ personal information or violate patients’ rights and therefore does not require ethical approval.

## 3 Discussion

Acupuncture is one of the most distinctive therapies in the system of Chinese medicine, and has become a universal medicine advocated by the World Health Organization (WHO) and applied in many countries and regions.^[17]^ Cerebral infarction, also known as ischemic stroke, is a common clinical cerebrovascular disease, accounting for 70% to 80% of all strokes, and lacunar infarction accounts for approximately 20% to 30% of all cerebral infarctions. Although lacunar infarcts are small in size, they can lead to neurological impairment and cognitive dysfunction.^[6,18,19]^ With easy operation, low cost, and low risk, acupuncture has been widely used in the treatment of lacunar cerebral infarction.

To date, a large number of relevant clinical trials have been published, but high-quality trials are still scanty. Compelling evidence is the key to embarking on a systematic review. To provide convincing evidence and better guide clinical practice, all operations in this review will be performed in accordance with Cochrane Handbook (5.2.0). The recognition of the role of acupuncture in the fight against LI is of great importance to patients. Therefore, this study aims to evaluate the safety and efficacy of acupuncture for LI patients in the recovery period through systematic review and meta-analysis, and to provide more methods for the treatment of LI.

## Author contributions

**Conceptualization:** Haoran Wang, Qiangsan Sun.

**Data curation:** Haoran Wang, Xiaoyan Fu, Jing Ju, Dan Meng.

**Investigation:** Haoran Wang, Jing Ju,.

**Methodology:** Haoran Wang, Xiaoyan Fu.

**Supervision:** Shengming Sun, Qiangsan Sun

**Validation:** Chenchen Guo, Hongling Jia.

**Visualization:** Haoran Wang.

**Writing – original draft:** Haoran Wang, Xiaoyan Fu.

**Writing – review & editing:** Hongling Jia, Qiangsan Sun.
